# Microsatellite markers for the biogeographically enigmatic sandmyrtle (*Kalmia buxifolia*, Phyllodoceae: Ericaceae)

**DOI:** 10.1002/aps3.11267

**Published:** 2019-06-05

**Authors:** Emily L. Gillespie, Tesa Madsen‐McQueen, Torsten Eriksson, Adam Bailey, Zack E. Murrell

**Affiliations:** ^1^ Department of Biological Sciences Butler University 4600 Sunset Avenue Indianapolis Indiana 46208 USA; ^2^ Department of Evolution, Ecology, and Organismal Biology University of California, Riverside 900 University Avenue Riverside California 92521 USA; ^3^ Department of Natural History University of Bergen Postboks 7800 5020 Bergen Norway; ^4^ Department of Biological Sciences Marshall University One John Marshall Drive Huntington West Virginia 25755 USA; ^5^ Department of Biology Appalachian State University 572 Rivers Street Boone North Carolina 28608 USA

**Keywords:** Ericaceae, *Kalmia buxifolia*, *Kalmia procumbens*, Phyllodoceae, sandmyrtle, species boundaries

## Abstract

**Premise:**

Microsatellite markers were developed for sandmyrtle, *Kalmia buxifolia* (Ericaceae), to facilitate phylogeographic studies in this taxon and possibly many of its close relatives.

**Methods and Results:**

Forty‐eight primer pairs designed from paired‐end Illumina MiSeq data were screened for robust amplification. Sixteen pairs were amplified again, but with fluorescently labeled primers to facilitate genotyping. Resulting chromatograms were evaluated for variability using three populations from Tennessee, North Carolina, and New Jersey, USA. Eleven primer pairs were reliable and polymorphic (mean 3.92 alleles), one was reliable but monomorphic, and four were not reliable. The markers exhibited lower heterozygosity (mean 0.246) than expected (mean 0.464). Cross‐amplification in the remaining nine *Kalmia* species exhibited a phylogenetic pattern, suggesting broad applicability of the markers across the genus.

**Conclusions:**

These microsatellite markers will be useful in population genetics and species boundaries studies of *K. buxifolia*,* K. procumbens*, and likely all other *Kalmia* species.

Sandmyrtle, *Kalmia buxifolia* (Bergius) Gift & Kron (Ericaceae), exhibits a disjunct distribution in eastern North America. Extant populations occur in the New Jersey Pinelands (NJP), the southern Appalachian Mountains (SAM), and the Sandhills/Cape Fear Arch (SCFA) region of the Carolinas, USA. There is no present‐day geographic connection between the NJP and SCFA populations, and very little between the SAM and SCFA populations. Despite relatively close geographic proximity, the SAM and SCFA populations are dissimilar in terms of habitat; SAM populations typically occur on rock outcrops, and SCFA populations occur in wet pinelands that are ecologically similar to the more distant NJP populations. Individual plants also vary morphologically, with SAM and NJP plants tending to be somewhat mat‐forming and SCFA plants tending to be taller and spindly. Over the past 150 years, experts have treated *K. buxifolia* as one (current consensus), two (Camp, 1938), or three (e.g., Small, 1933) species based on a combination of morphology and habitat.

The phylogeny of the North American genus *Kalmia* L. (Phyllodoceae: Ericaceae) is well resolved with four clades of two to three species each (fig. 3 in Gillespie and Kron, 2013). Clade 1 includes the sandy habitat specialists hairy laurel, *K. hirsuta* Walter (narrowly distributed and centered in northern Florida, USA), and Cuban kalmia, *K. ericoides* C. Wright ex Griseb. (western Cuba), as well as mountain laurel, *K. latifolia* L. (eastern North America). Clade 2 is composed of the circumboreal alpine azalea, *K. procumbens* (L.) Gift, Kron & P. F. Stevens ex Galasso, Banfi & F. Conti, which is strongly supported as the sister of *K. buxifolia*. Clade 3 includes sheep laurel, *K. angustifolia* L., Carolina sheep laurel, *K. carolina* Small, and whitewicky, *K. cuneata* Michx., which have large to small ranges, respectively, within deciduous forests of eastern North America (with *K. cuneata* specializing in pocosin habitats). Within Clade 4, bog laurel, *K. polifolia* Wangenh., is found across Canada and in the Upper Midwest, USA, but slightly overlaps in distribution with western bog laurel, *K. microphylla* (Hook.) A. Heller, in the Pacific Northwest, USA, and upper Rocky Mountains, Canada. Most non‐cultivated *Kalmia*, including *K. buxifolia*, are reportedly diploid based on flow cytometry, with tetraploidy being consistently detected in *K. angustifolia* and *K. polifolia* (Gillooly and Ranney, 2015). Jaynes (1969) found that the diploid *Kalmia* species were uniformly *n* = 12 based on chromosome counts, but *K. buxifolia* was at that time still considered a separate genus (*Leiophyllum* (Pers.) R. Hedw.), and was not included in that study. However, Hagerup (1928) found that both *K. buxifolia* (then *L. buxifolium* (Bergius) Elliott) and *K. procumbens* (then *Loiseleuria procumbens* (L.) Desv.) are both *n* = 12. The morphological and genetic structure of *K. buxifolia*, as well as its phylogeographic history, are poorly understood at the population level. No molecular markers currently exist for fine‐scale study within *Kalmia*. Development of microsatellite markers focused on two goals: (1) to develop markers specifically for investigation of genetic patterns on a landscape scale across the entire disjunct distribution of *K. buxifolia*, and (2) to preliminarily investigate the utility of *K. buxifolia* markers across all remaining *Kalmia* species, within a phylogenetic context.

## METHODS AND RESULTS

Details of all bioinformatics, screening, and genotyping protocols followed Kasireddy et al. (2018). Paired‐end MiSeq Illumina sequencing was performed on a single *K. buxifolia* cetyltrimethylammonium bromide (CTAB) DNA extraction (Doyle and Doyle, 1987) (Appendix 1) with CsCl_2_ purification modified from Palmer (1986). The raw sequence reads were trimmed in Geneious 11.1.5 (Kearse et al., 2012) and explored using MSATCOMMANDER (Faircloth, 2008). Out of 285,035 reads that contained microsatellites, 44,731 allowed for primer design and were considered for amplification screening.

An amplification screen of 48 primer pairs was carried out using seven *K. buxifolia* individuals (Appendix 1) extracted using a QIAGEN Plant Mini Kit (QIAGEN, Hilden, Germany) with modifications following Drábková et al. (2002). Sixteen primer pairs (Table 1), including a diversity of repeat motifs (*n* = 8 hexamer, *n* = 4 pentamer, *n* = 3 tetramer, and *n* = 2 trimer) representing putatively independent loci, produced single amplicons in the anticipated size range.

**Table 1:** Characteristics of 16 microsatellite primer pairs investigated for *Kalmia buxifolia*

Locus	Primer sequences (5′–3′)^a^	Fluorescent dye	Repeat motif	*T*_a_ (°C)	Allele size range (bp)	GenBank accession no.
KBUX002^b^	F: ACAAACCAAGACGTAAACAACC	PET	(AAAAAT)_7_	59.1	264	MK333440
	R: GTTTGCTGATTCGTGTGCCTC					
KBUX003	F: CCCATTTACCAGCCTAAACCAC	NED	(AAAACT)_7_	59.7	184–214	MK333439
	R: GTTTCGATGGTGGTGAAGATGGC					
KBUX004^c^	F: AGAGACGGAAACATGGACGG	NED	(AAAAG)_6_	60.7	NA	MK333438
	R: GTTTCGCACGTGAGCTCCTTATG					
KBUX005	F: GTTTGCACCCTTCCGATTTACC	PET	(AAAAT)_6_	59.6	260–285	MK333437
	R: TAAGGCGGCCCAACTTCTAG					
KBUX008^c^	F: GGATTCATGTAGCCGACCC	6‐FAM	(AAACC)_6_	58.4	NA	MK333436
	R: GTTTGCACATCACCATAATATTGCG					
KBUX009	F: CATGGATCGGTTTGGATTTGG	6‐FAM	(AAACCC)_9_	58.4	90–150	MK333435
	R: GTTTAGCAAAGTATCCGGCCTAAC					
KBUX010	F: ACCAAACCAAGCCAAGACAC	PET	(AAAG)_6_	59.6	142–258	MK333444
	R: GTTTACAGTGTGAAAGGAAGAAGCTG					
KBUX015	F: GTCTGCTGCTCTGTTCTTCG	VIC	(AACACG)_6_	59.4	183–219	MK333443
	R: GTTTACTTTCAATTGTCTCCCGCC					
KBUX016	F: GTTTGACTTGAAGAGCGTGGACC	NED	(AACC)_8_	60.6	170–210	MK333434
	R: CTGTTCTCGCTGCAACACTG					
KBUX020^c^	F: GATATTTCAAGTGTGGTGTGGC	PET	(AACCG)_8_	58.9	NA	MK333433
	R: GTTTAACCGATCCAAACCGAAGTG					
KBUX021	F: AAGAACTGTTTGCGTGACGG	VIC	(AACGAG)_6_	59.2	136–166	MK333432
	R: GTTTCTGACGACAAGGACAAGG					
KBUX027	F: GCAACAAGGATCCGAGTCTC	6‐FAM	(AAGGAG)_11_	59.3	162–192	MK333431
	R: GTTTGCTCAAAGTATTCATCCCGC					
KBUX034^c^	F: ACACAACTTGAGGGAGGGTC	VIC	(AATG)_8_	59.7	NA	MK333430
	R: GTTTACGTGGTATGCTACTCCCTC					
KBUX036	F: CGATTAGCAACGTCGAGTGG	NED	(ACACCG)_7_	59.9	172–214	MK333429
	R: GTTTAGAATTGCCGTGTCCGTG					
KBUX039	F: GTTTGCTGGTTGATGCGGTG	6‐FAM	(ACC)_8_	60.0	130–154	MK333442
	R: CAGCCACCGACAAAGACATC					
KBUX047	F: GAATTCTGTTCGACCGCCTC	VIC	(CCG)_10_	60.0	170–191	MK333441
	R: GTTTCTCAACGTCCCTGATCTGC					

Note

*T*_a_ = annealing temperature.

a PIG‐tail sequence is underlined on primers.

b Monomorphic marker.

c Markers that genotyped inconsistently or poorly.

A follow‐up polymorphism screen of the 16 primer pairs involved a second round of PCR for 67 individuals from three maximally spaced populations from Tennessee (SAM), North Carolina (SCFA), and New Jersey (NJP) (Appendix 1). Individuals collected were at least several meters apart, as clonality in this species has not been clarified. These PCRs also incorporated fluorescently tagged (6‐FAM, VIC, NED, or PET; Life Technologies, Grand Island, New York, USA) M13 universal primers to facilitate separate visualization of chromatograms from pooled amplicons. Resulting chromatograms were manually scored using Geneious 11.1.5. As in Kasireddy et al. (2018), we employed strict quality criteria for identifying peaks generally and heterozygotes specifically (3000 relative fluorescence units [RFUs] and similar peak height, respectively). GenAlEx 6.503 (Peakall and Smouse, 2006, 2012) was used to characterize the resulting genotypes, including a test to detect deviation from Hardy–Weinberg equilibrium (HWE), a multilocus matches analysis (Peakall and Smouse, 2006, 2012) to explore clonality, and a principal coordinate analysis (Orloci, 1978) to determine the degree to which the markers collectively separated populations.

Twelve loci revealed chromatograms with no more than two peaks, indicating diploidy. Four markers (KBUX004, KBUX008, KBUX020, and KBUX034) genotyped inconsistently or poorly, and were abandoned. The 12 successful markers exhibited 1–11 alleles across three populations (mean 3.92) (Table 2). One marker (KBUX002) was monomorphic in all three populations. Observed heterozygosity ranged from 0.000 to 0.762 (mean 0.246), with all three populations exhibiting lower than expected heterozygosity. All 11 polymorphic loci failed to meet the expectations of HWE in at least one population. Of these, four loci (KBUX005, KBUX021, KBUX036, and KBUX047) failed to meet HWE expectations in all three populations. Genetic distance followed by principal coordinate analysis demonstrated that the 11 polymorphic loci clearly distinguish populations, with the first three axes explaining 37.18% of the variation. A multilocus matches analysis of 11 polymorphic loci revealed only two identical individuals (both from Tennessee, USA), suggesting very limited clonality and/or successful sampling of distinct individuals.

**Table 2:** Descriptive statistics for 12 microsatellite loci in three populations of *Kalmia buxifolia*.^a^

Locus	NJP: Ocean Co., NJ (*N* = 24)				SCFA: Brunswick Co., NC (*N* = 21)				SAM: Sevier Co., TN (*N* = 22)			
	*A*	*H*_o_	*H*_e_	HWE^b^	*A*	*H*_o_	*H*_e_	HWE^b^	*A*	*H*_o_	*H*_e_	HWE^b^
KBUX002	1	0.000	0.000	M	1	0.000	0.000	M	1	0.000	0.000	M
KBUX003	3	0.292	0.442	NS	5	0.368	0.708	*	0	NA	NA	NA
KBUX005	4	0.250	0.640	**	6	0.286	0.397	***	2	0.136	0.416	**
KBUX009	8	0.708	0.754	NS	4	0.105	0.393	***	11	0.682	0.812	NS
KBUX010	1	0.000	0.000	M	5	0.045	0.286	***	1	0.000	0.000	M
KBUX015	2	0.222	0.500	NS	4	0.136	0.721	***	3	0.158	0.193	**
KBUX016	5	0.125	0.572	***	5	0.000	0.587	***	4	0.136	0.209	NS
KBUX021	4	0.167	0.705	***	6	0.263	0.802	***	5	0.091	0.683	***
KBUX027	5	0.417	0.488	NS	4	0.500	0.551	NS	4	0.318	0.346	*
KBUX036	3	0.375	0.624	***	3	0.095	0.540	***	4	0.318	0.499	***
KBUX039	3	0.417	0.518	NS	6	0.762	0.721	NS	4	0.095	0.511	***
KBUX047	5	0.542	0.699	***	4	0.143	0.684	***	5	0.591	0.719	***
Mean	3.67	0.302	0.495		4.42	0.225	0.532		3.67	0.210	0.366	

Note

*A* = number of alleles detected across all individuals; *H*_e_ = expected heterozygosity; *H*_o_ = observed heterozygosity; HWE = Hardy–Weinberg equilibrium; *N* = number of individuals.

a Locality and voucher information are provided in Appendix 1.

^b^Asterisks (*) indicate statistically significant deviation from HWE (*P < 0.05; **P < 0.01; ***P < 0.001). M = monomorphic marker; NS = not statistically significant.

Cross‐amplification of 12 primer pairs was conducted within a phylogenetic context, following Gillespie and Kron (2013). Five *K. procumbens* individuals (Clade 2, which includes *K. buxifolia*) and one individual each of *K. angustifolia*,* K. carolina*,* K. cuneata* (Clade 3); *K. ericoides*,* K. hirsuta*,* K. latifolia* (Clade 1); and *K. microphylla* and *K. polifolia* (Clade 4) were included. KBUX009 and KBUX039 failed to amplify in any other taxon, including *K. procumbens*, the nearest relative of *K. buxifolia*. KBUX036 amplified only in *K. procumbens* and was monomorphic. Seven markers amplified well in Clade 3 and Clade 4 (with an eighth amplifying well in Clade 4), whereas five markers amplified in Clade 1 (excluding *K. hirsuta*, in which three markers amplified) (Table 3).

**Table 3:** Cross‐amplification of 12 primer pairs within *Kalmia*.^a^,^b^,^c^

Species	Clade 1			Clade 2	Clade 3			Clade 4	
	Keri	Khir	Klat	Kpro	Kang	Kcar	Kcun	Kmic	Kpol
KBUX002	264	—	264	264–269	264	264	259	264	264
KBUX003	—	—	196	184–190	—	—	—	—	—
KBUX005	265	—	260	255–265	—	—	—	255	260
KBUX009	—	—	—	—	—	—	—	—	—
KBUX010	—	—	150	138–154	146	142	150	150	146
KBUX015	189	189	177	177–213	195	177	183	195	219
KBUX016	—	‐—	174	178–186	182	182	178	182	186
KBUX021	148	154	154	160–172	166	166	154	166	178
KBUX027	—	—	—	156–180	168	162	162	180	186
KBUX036	—	—	—	142	—	—	—	—	—
KBUX039	—	—	—	—	—	—	—	—	—
KBUX047	176	182	185	173–182	185	182	176	176	173

Note

— = no observable amplification; Keri *= Kalmia ericoides*; Khir *= Kalmia hirsuta*; Klat *= Kalmia latifolia*; Kpro = *Kalmia procumbens*; Kang = *Kalmia angustifolia*; Kcar = *Kalmia carolina*; Kcun *= Kalmia cuneata*; Kmic *= Kalmia microphylla*; Kpol *= Kalmia polifolia*.

a Locality and voucher information for outgroup representatives are given in Appendix 1.

b Allele size range is given if multiple individuals were sampled and the marker was polymorphic.

c All outgroup taxa are *N* = 1, except *K. procumbens* (*N* = 5). Clades 1–4 follow the phylogeny of *Kalmia* from Gillespie and Kron (2013).

## CONCLUSIONS

The markers reported here will be useful in planned population and phylogeography studies across the range of *K. buxifolia* and will most likely be useful within *K. procumbens*. Amplification of the markers in related species was generally successful, with somewhat less success in the Clade 1 sandy habitat specialists of Florida and Cuba.

## AUTHOR CONTRIBUTIONS

E.L.G., T.M‐M., and Z.E.M. determined the sampling strategy and collected *Kalmia buxifolia* populations in the United States. E.L.G. and A.B. conducted all genetic work, and T.E. sampled the nearest relative, *K. procumbens*, in Norway. E.L.G. collected most other *Kalmia* outgroups. Z.E.M. conceptualized and managed the project framework. E.L.G. and Z.E.M. labs financed the project. E.L.G. conducted analyses, and all co‐authors assisted with manuscript preparation.

## Data Availability

The raw sequence reads are deposited in the National Center for Biotechnology Information (NCBI; GenBank Sequence Read Archive accession no. SRP173774). Sequence information for the developed primers has been deposited to NCBI; accession numbers are provided in Table 1.

## APPENDIX 1 Voucher information for individuals included in this study.

**Table:**

Species	Voucher (Herbarium)	Geographic coordinates		Elevation (m)	State (Country)	County	*N*
		Latitude	Longitude				
*Kalmia buxifolia*	Kron 2067 (WFU)^a^	35.65	−83.43	1980	Tennessee (USA)	Sevier	1
*Kalmia buxifolia*	Gillespie 17‐007 (BUT)^b^	39.82	−74.97	21	New Jersey (USA)	Ocean	24
*Kalmia buxifolia*	Madsen‐McQueen 17‐011 (BOON)^b^	34.65	−83.44	1994	Tennessee (USA)	Sevier	22
*Kalmia buxifolia*	Madsen‐McQueen 17‐021 (BOON)^b^	34.00	−78.04	11	North Carolina (USA)	Brunswick	21
*Kalmia procumbens*	Eriksson 1086 (BUT)^c^	60.55	6.07	840	Hordaland (Norway)	NA	5
*Kalmia angustifolia*	Gillespie 17‐020 (BUT)^c^	39.77	−74.41	33	New Jersey (USA)	Ocean	1
*Kalmia carolina*	Gillespie 13‐147 (BUT)^c^	38.49	−81.14	893	North Carolina (USA)	Alleghany	1
*Kalmia cuneata*	Gillespie 07‐003 (WFU)^c^	34.64	−78.60	12	North Carolina (USA)	Bladen	1
*Kalmia ericoides*	Abbot 18854 (FLAS)^c^	22.12	−84.00	11	Pinar del Rio (Cuba)	Guane	1
*Kalmia hirsuta*	Gillespie 13‐123 (BUT)^c^	30.71	−83.04	41	Georgia (USA)	Echols	1
*Kalmia latifolia*	Gillespie 13‐026 (BUT)^c^	37.37	−80.52	1224	Virginia (USA)	Giles	1
*Kalmia microphylla*	Gillespie 06‐020 (WFU)^c^	40.49	−121.42	2042	California (USA)	Shasta	1
*Kalmia polifolia*	Poindexter 07‐471 (BOON)^c^	45.32	−80.00	236	Wisconsin (USA)	Marinette	1

Note

BOON = I. W. Carpenter, Jr. Herbarium (Appalachian State University); BUT = Friesner Herbarium (Butler University); *N* = number of individuals; WFU = Wake Forest University Herbarium.

a Voucher for Illumina sequencing.

b Voucher for marker development (separate collection effort).

c Voucher for cross‐amplification.
